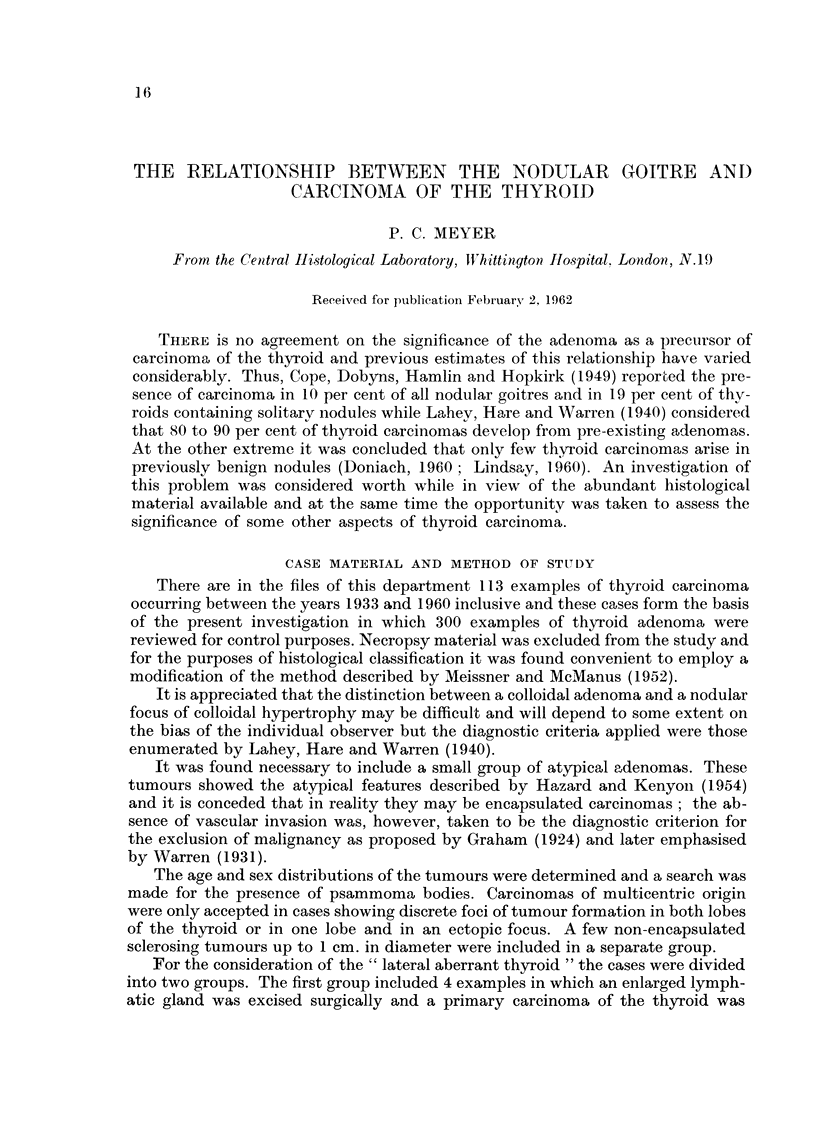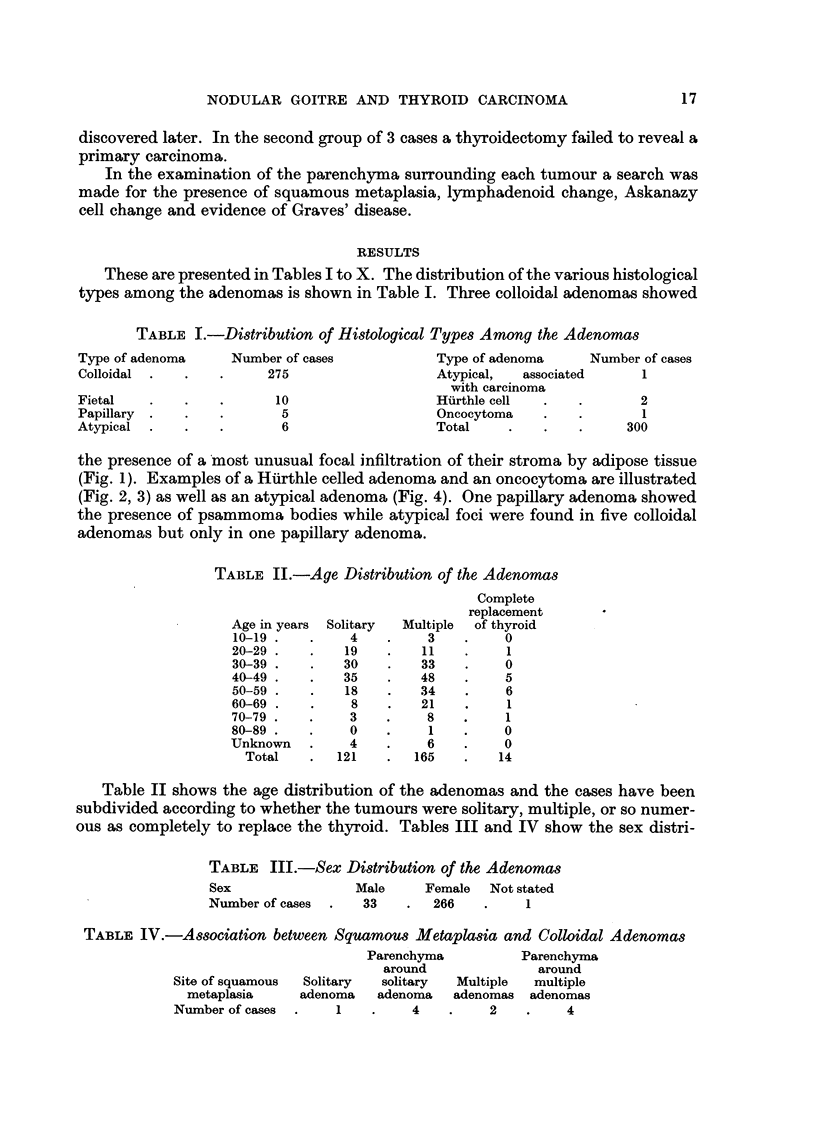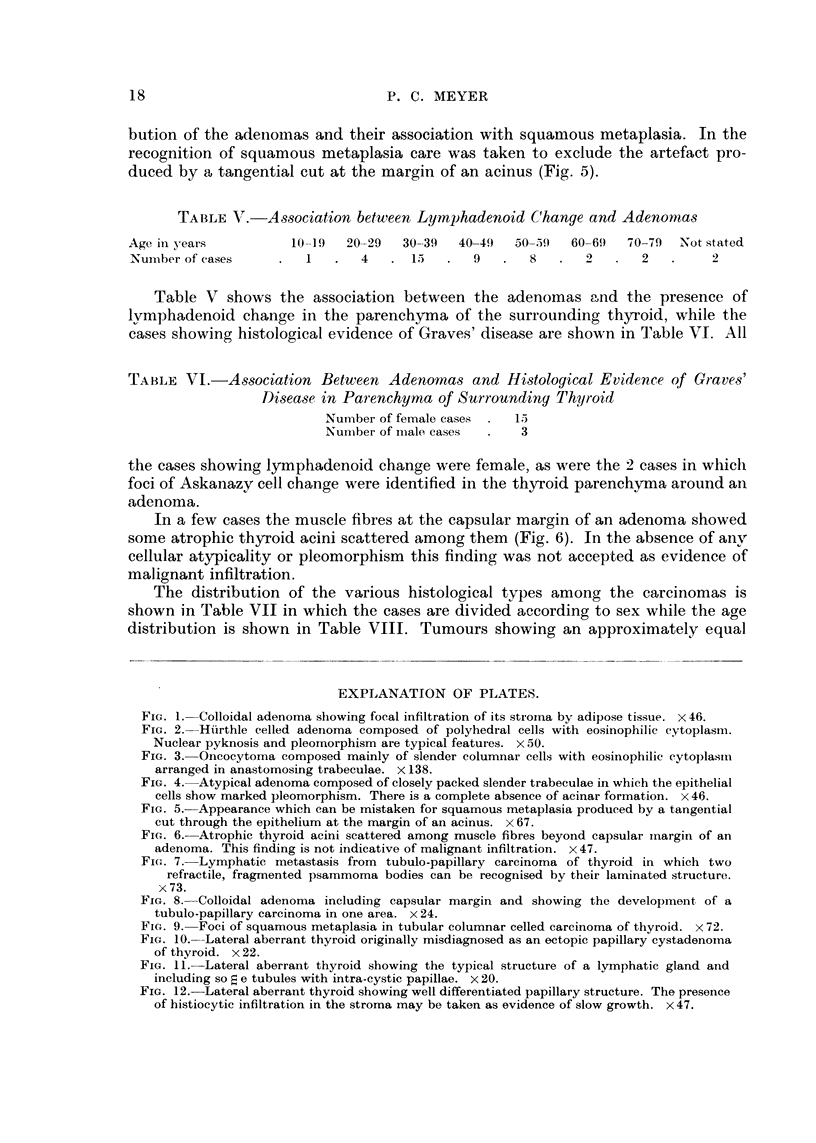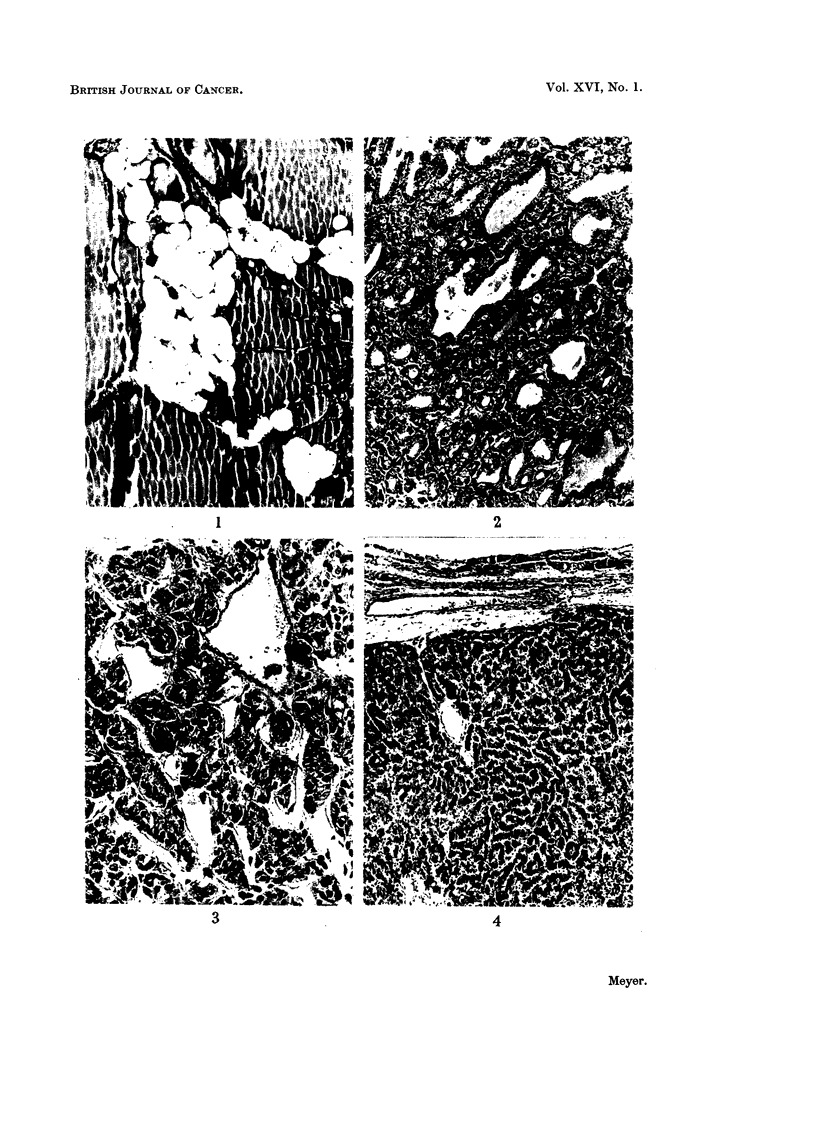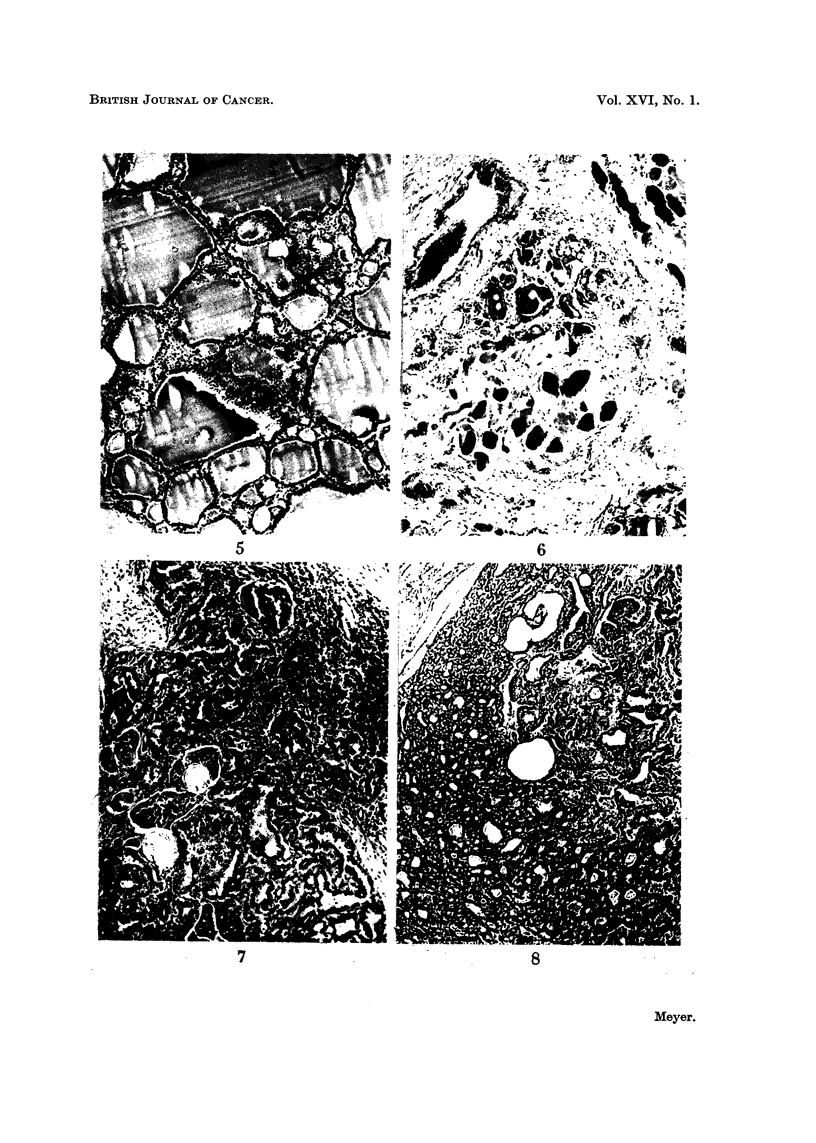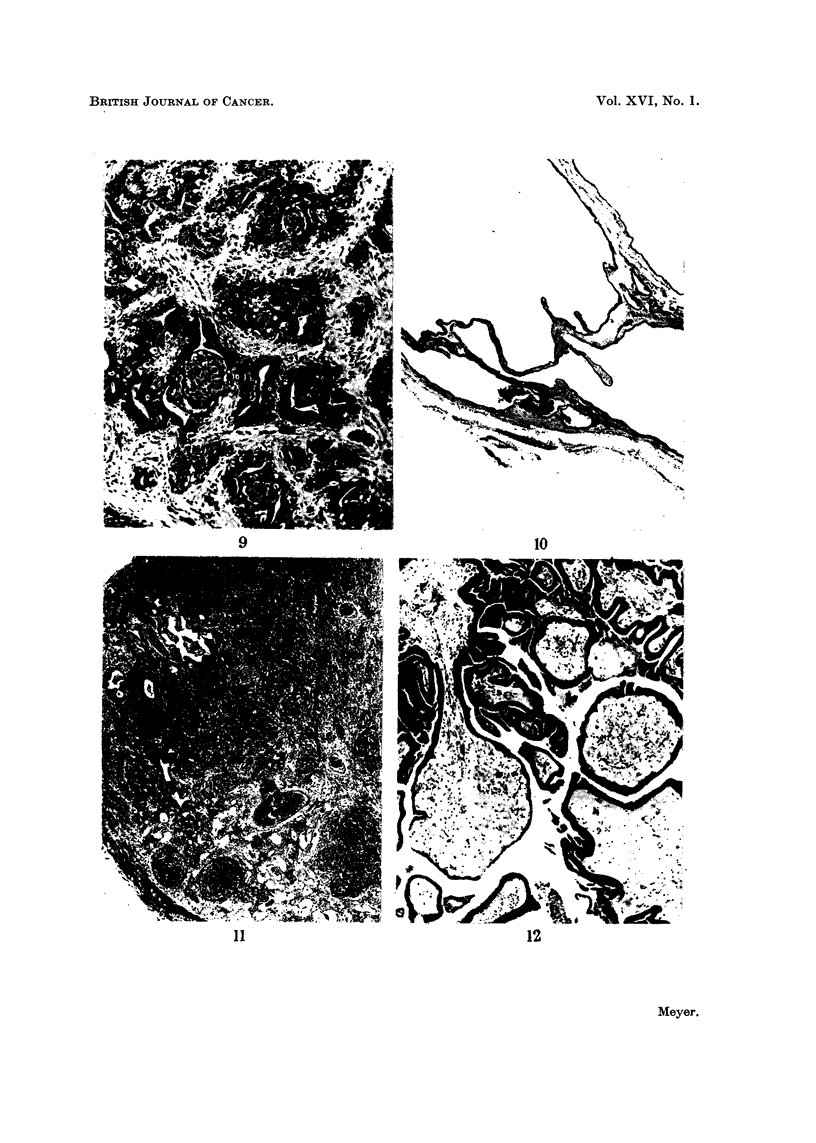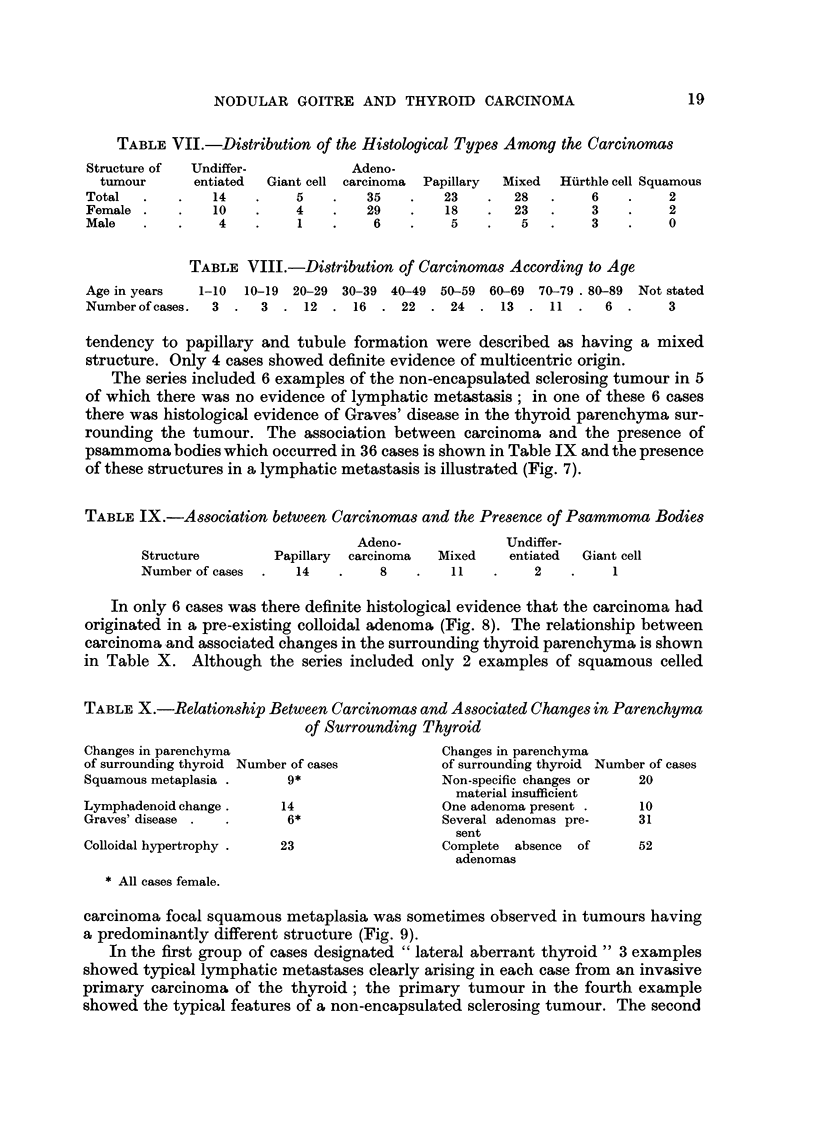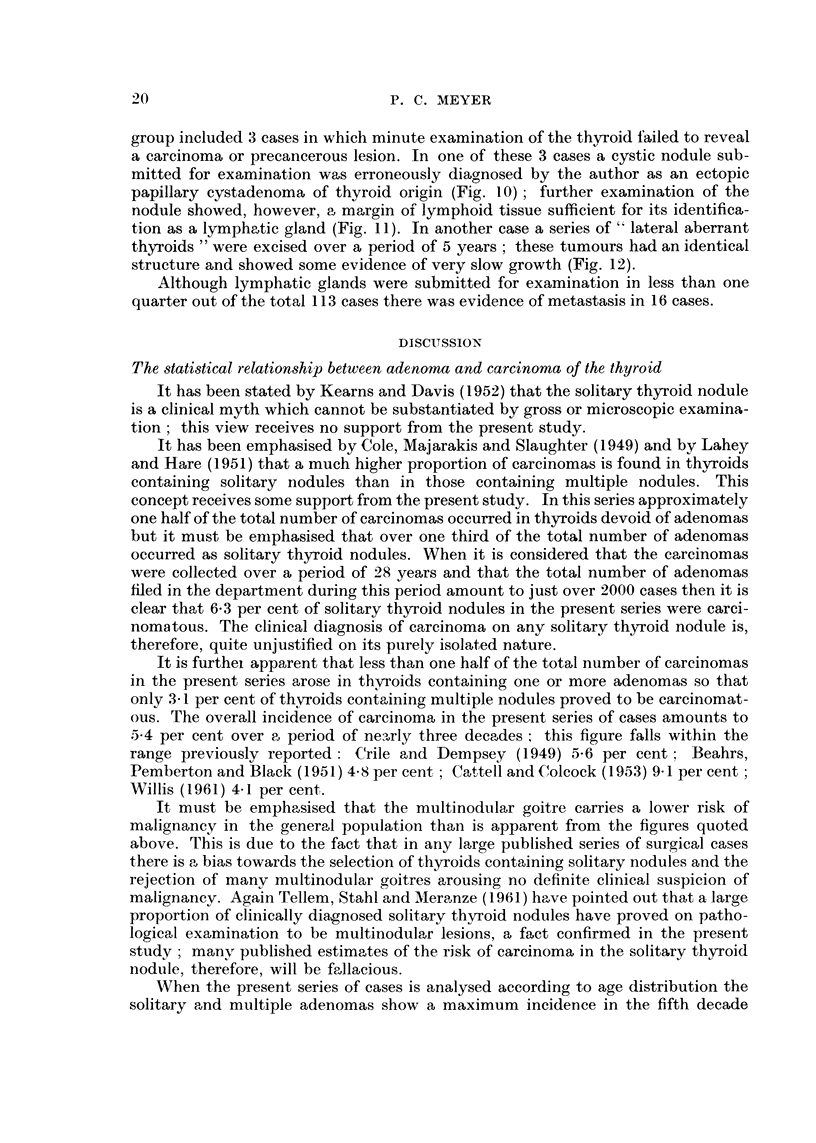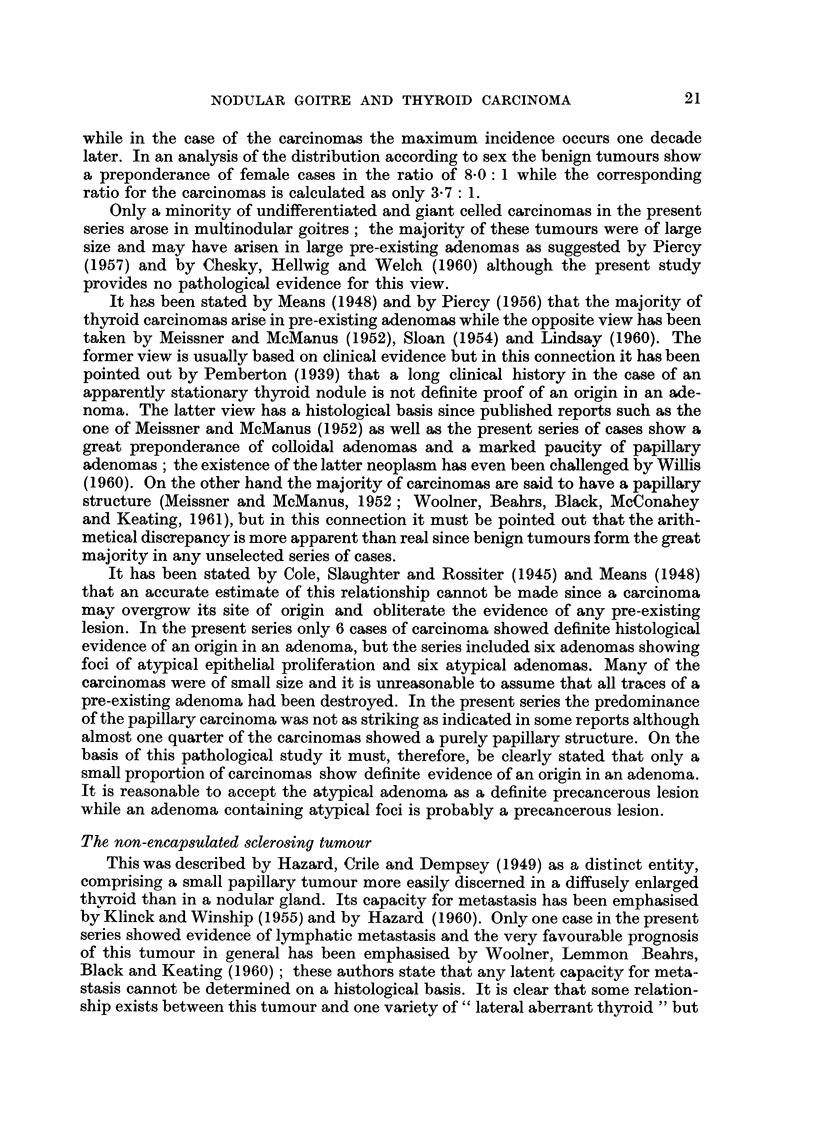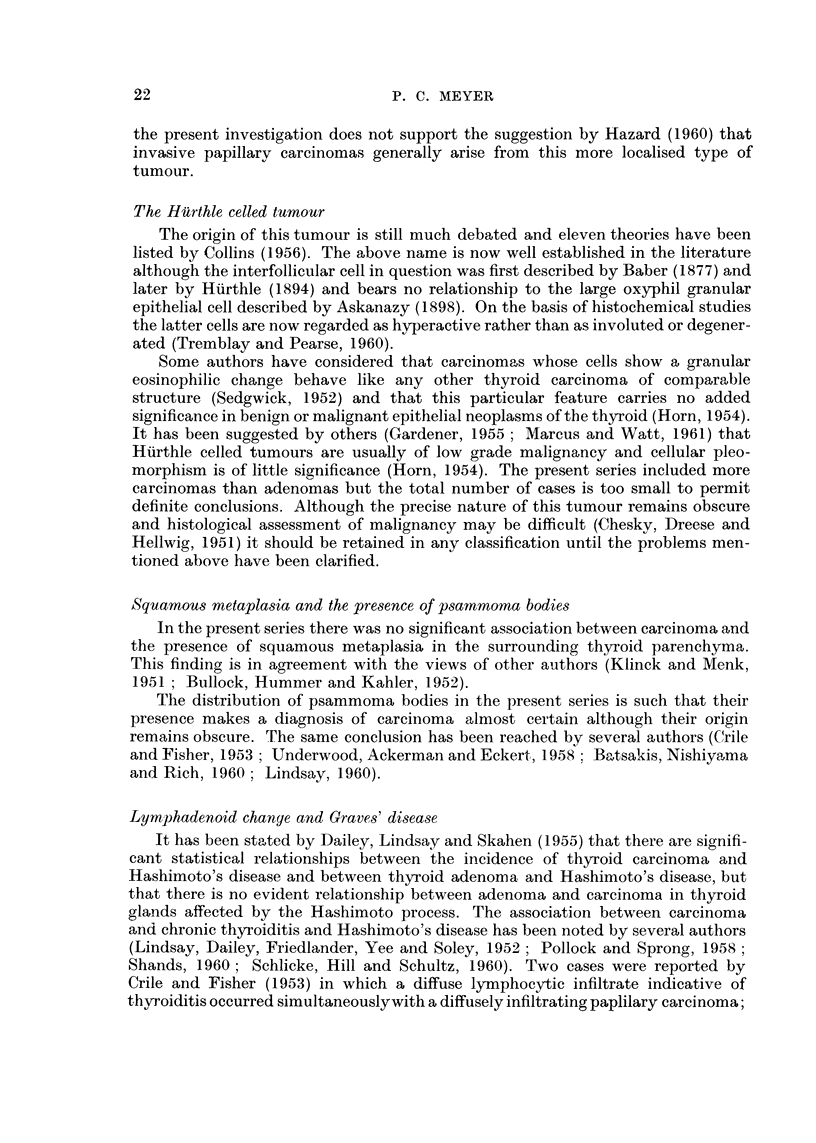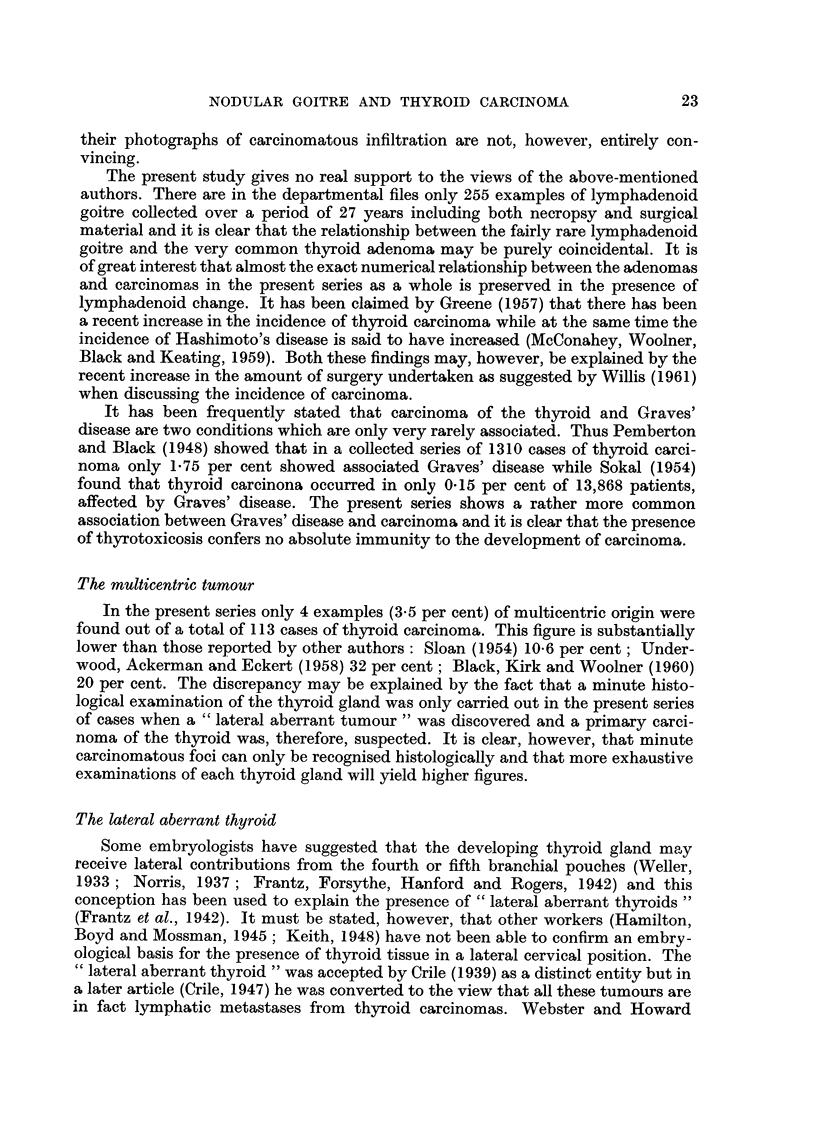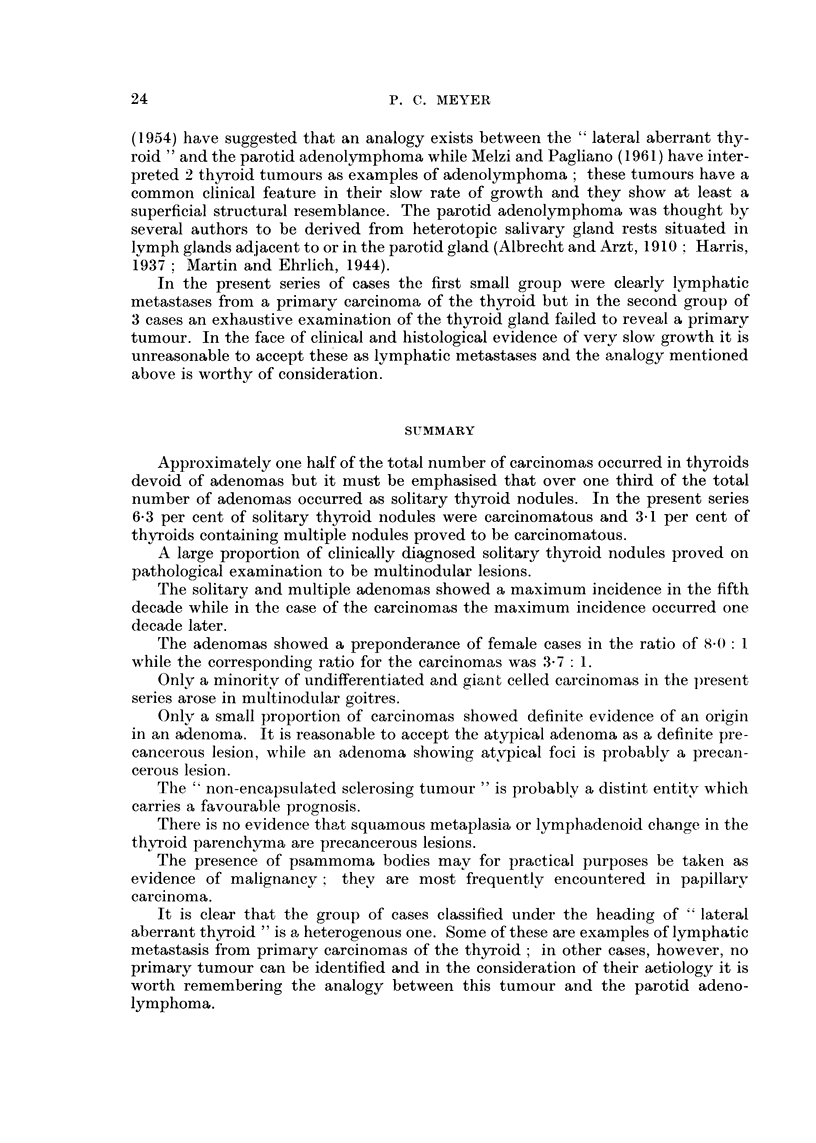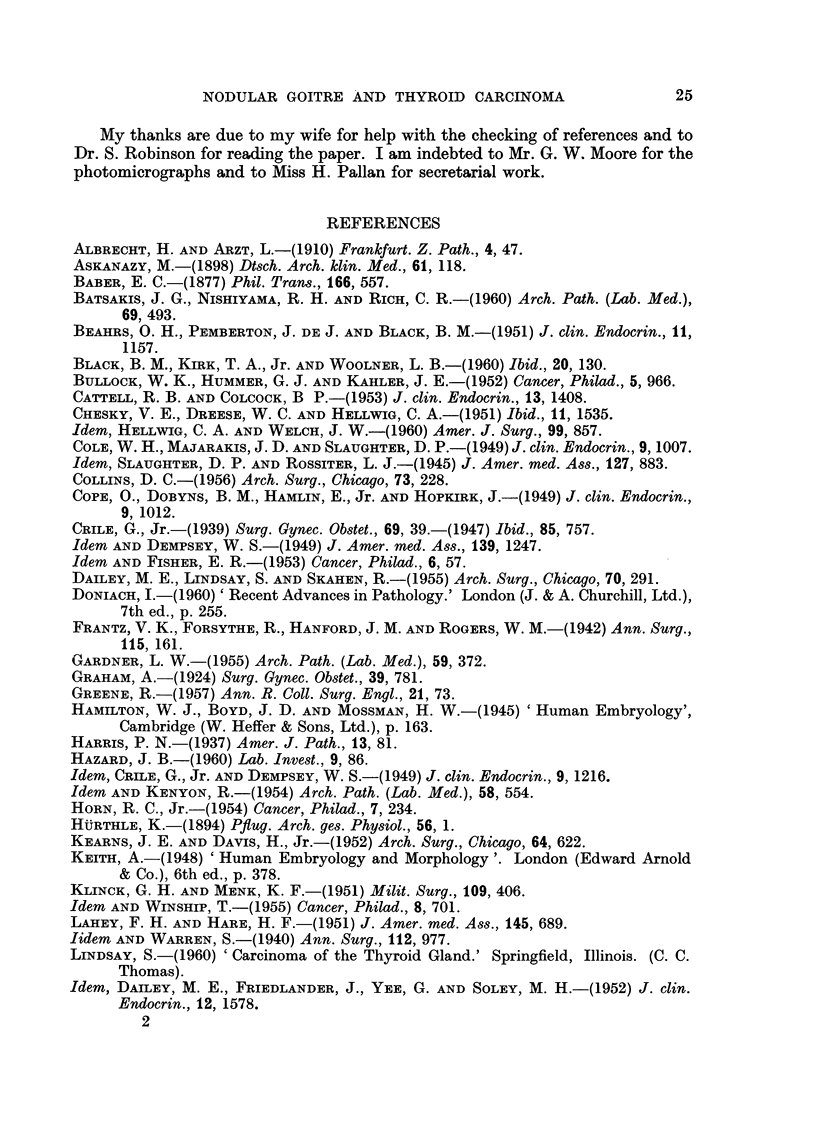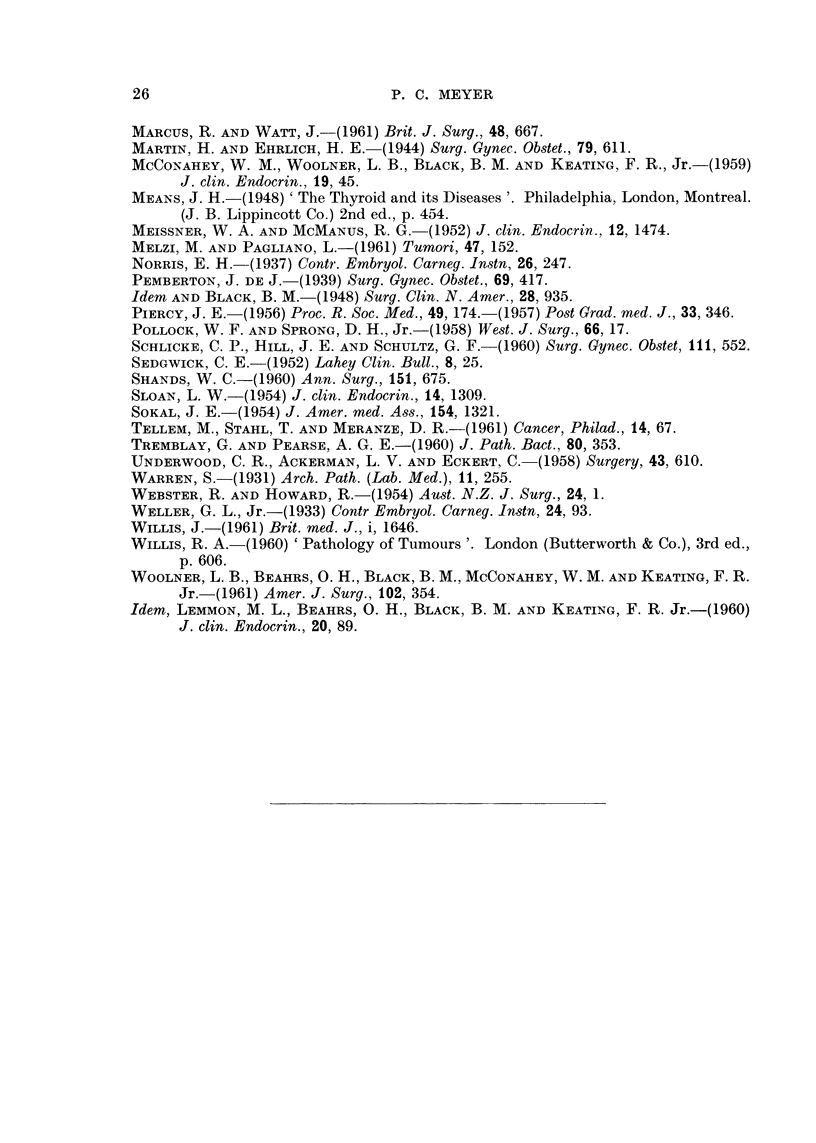# The Relationship Between the Nodular Goitre and Carcinoma of the Thyroid

**DOI:** 10.1038/bjc.1962.3

**Published:** 1962-03

**Authors:** P. C. Meyer

## Abstract

**Images:**


### THE RELATIONSHIP BETWEEN THE NODULAR GOITRE AND CARCINOMA OF THE THYROID

P. C. MEYER

*From the Central Histological Laboratory, Whittington Hospital, London, N.19*

Received for publication February 2, 1962

THERE is no agreement on the significance of the adenoma as a precursor of carcinoma of the thyroid and previous estimates of this relationship have varied considerably. Thus, Cope, Dobyns, Hamlin and Hopkirk (1949) reported the presence of carcinoma in 10 per cent of all nodular goitres and in 19 per cent of thyroids containing solitary nodules while Lahey, Hare and Warren (1940) considered that 80 to 90 per cent of thyroid carcinomas develop from pre-existing adenomas. At the other extreme it was concluded that only few thyroid carcinomas arise in previously benign nodules (Doniach, 1960 ; Lindsay, 1960). An investigation of this problem was considered worth while in view of the abundant histological material available and at the same time the opportunity was taken to assess the significance of some other aspects of thyroid carcinoma.

### CASE MATERIAL AND METHOD OF STUDY

There are in the files of this department 113 examples of thyroid carcinoma occurring between the years 1933 and 1960 inclusive and these cases form the basis of the present investigation in which 300 examples of thyroid adenoma were reviewed for control purposes. Necropsy material was excluded from the study and for the purposes of histological classification it was found convenient to employ a modification of the method described by Meissner and McManus (1952).

It is appreciated that the distinction between a colloidal adenoma and a nodular focus of colloidal hypertrophy may be difficult and will depend to some extent on the bias of the individual observer but the diagnostic criteria applied were those enumerated by Lahey, Hare and Warren (1940).

It was found necessary to include a small group of atypical adenomas. These tumours showed the atypical features described by Hazard and Kenyon (1954) and it is conceded that in reality they may be encapsulated carcinomas ; the absence of vascular invasion was, however, taken to be the diagnostic criterion for the exclusion of malignancy as proposed by Graham (1924) and later emphasised by Warren (1931).

The age and sex distributions of the tumours were determined and a search was made for the presence of psammoma bodies. Carcinomas of multicentric origin were only accepted in cases showing discrete foci of tumour formation in both lobes of the thyroid or in one lobe and in an ectopic focus. A few non-encapsulated sclerosing tumours up to 1 cm. in diameter were included in a separate group.

For the consideration of the " lateral aberrant thyroid " the cases were divided into two groups. The first group included 4 examples in which an enlarged lymphatic gland was excised surgically and a primary carcinoma of the thyroid was discovered later. In the second group of 3 cases a thyroidectomy failed to reveal a primary carcinoma.

In the examination of the parenchyma surrounding each tumour a search was made for the presence of squamous metaplasia, lymphadenoid change, Askanazy cell change and evidence of Graves' disease.

### RESULTS

These are presented in Tables I to X. The distribution of the various histological types among the adenomas is shown in Table I. Three colloidal adenomas showed

TABLE I.—*Distribution of Histological Types Among the Adenomas*

| Type of adenoma | Number of cases | Type of adenoma | Number of cases |
|---|---|---|---|
| Colloidal | 275 | Atypical, associated with carcinoma | 1 |
| Fietal | 10 | Hürthle cell | 2 |
| Papillary | 5 | Oncocytoma | 1 |
| Atypical | 6 | Total | 300 |

the presence of a most unusual focal infiltration of their stroma by adipose tissue (Fig. 1). Examples of a Hürthle celled adenoma and an oncocytoma are illustrated (Fig. 2, 3) as well as an atypical adenoma (Fig. 4). One papillary adenoma showed the presence of psammoma bodies while atypical foci were found in five colloidal adenomas but only in one papillary adenoma.

TABLE II.—*Age Distribution of the Adenomas*

| Age in years | Solitary | Multiple | Complete replacement of thyroid |
|---|---|---|---|
| 10–19 | 4 | 3 | 0 |
| 20–29 | 19 | 11 | 1 |
| 30–39 | 30 | 33 | 0 |
| 40–49 | 35 | 48 | 5 |
| 50–59 | 18 | 34 | 6 |
| 60–69 | 8 | 21 | 1 |
| 70–79 | 3 | 8 | 1 |
| 80–89 | 0 | 1 | 0 |
| Unknown | 4 | 6 | 0 |
| Total | 121 | 165 | 14 |

Table II shows the age distribution of the adenomas and the cases have been subdivided according to whether the tumours were solitary, multiple, or so numerous as completely to replace the thyroid. Tables III and IV show the sex distri-

TABLE III.—*Sex Distribution of the Adenomas*

| Sex | Male | Female | Not stated |
|---|---|---|---|
| Number of cases | 33 | 266 | 1 |

TABLE IV.—*Association between Squamous Metaplasia and Colloidal Adenomas*

| Site of squamous metaplasia | Solitary adenoma | Parenchyma around solitary adenoma | Multiple adenomas | Parenchyma around multiple adenomas |
|---|---|---|---|---|
| Number of cases | 1 | 4 | 2 | 4 |

bution of the adenomas and their association with squamous metaplasia. In the recognition of squamous metaplasia care was taken to exclude the artefact produced by a tangential cut at the margin of an acinus (Fig. 5).

TABLE V.—*Association between Lymphadenoid Change and Adenomas*

| Age in years | 10–19 | 20–29 | 30–39 | 40–49 | 50–59 | 60–69 | 70–79 | Not stated |
|---|---|---|---|---|---|---|---|---|
| Number of cases | 1 | 4 | 15 | 9 | 8 | 2 | 2 | 2 |

Table V shows the association between the adenomas and the presence of lymphadenoid change in the parenchyma of the surrounding thyroid, while the cases showing histological evidence of Graves' disease are shown in Table VI. All

TABLE VI.—*Association Between Adenomas and Histological Evidence of Graves' Disease in Parenchyma of Surrounding Thyroid*

| | |
|---|---|
| Number of female cases | 15 |
| Number of male cases | 3 |

the cases showing lymphadenoid change were female, as were the 2 cases in which foci of Askanazy cell change were identified in the thyroid parenchyma around an adenoma.

In a few cases the muscle fibres at the capsular margin of an adenoma showed some atrophic thyroid acini scattered among them (Fig. 6). In the absence of any cellular atypicality or pleomorphism this finding was not accepted as evidence of malignant infiltration.

The distribution of the various histological types among the carcinomas is shown in Table VII in which the cases are divided according to sex while the age distribution is shown in Table VIII. Tumours showing an approximately equal

---

EXPLANATION OF PLATES.

FIG. 1.—Colloidal adenoma showing focal infiltration of its stroma by adipose tissue. ×46.

FIG. 2.—Hürthle celled adenoma composed of polyhedral cells with eosinophilic cytoplasm. Nuclear pyknosis and pleomorphism are typical features. ×50.

FIG. 3.—Oncocytoma composed mainly of slender columnar cells with eosinophilic cytoplasm arranged in anastomosing trabeculae. ×138.

FIG. 4.—Atypical adenoma composed of closely packed slender trabeculae in which the epithelial cells show marked pleomorphism. There is a complete absence of acinar formation. ×46.

FIG. 5.—Appearance which can be mistaken for squamous metaplasia produced by a tangential cut through the epithelium at the margin of an acinus. ×67.

FIG. 6.—Atrophic thyroid acini scattered among muscle fibres beyond capsular margin of an adenoma. This finding is not indicative of malignant infiltration. ×47.

FIG. 7.—Lymphatic metastasis from tubulo-papillary carcinoma of thyroid in which two refractile, fragmented psammoma bodies can be recognised by their laminated structure. ×73.

FIG. 8.—Colloidal adenoma including capsular margin and showing the development of a tubulo-papillary carcinoma in one area. ×24.

FIG. 9.—Foci of squamous metaplasia in tubular columnar celled carcinoma of thyroid. ×72.

FIG. 10.—Lateral aberrant thyroid originally misdiagnosed as an ectopic papillary cystadenoma of thyroid. ×22.

FIG. 11.—Lateral aberrant thyroid showing the typical structure of a lymphatic gland and including so[illegible]e tubules with intra-cystic papillae. ×20.

FIG. 12.—Lateral aberrant thyroid showing well differentiated papillary structure. The presence of histiocytic infiltration in the stroma may be taken as evidence of slow growth. ×47.

1 2 3 4

Meyer.

5

6

7

8

Meyer.

9 10 11 12

Meyer.

TABLE VII.—*Distribution of the Histological Types Among the Carcinomas*

| Structure of tumour | Undifferentiated | Giant cell | Adenocarcinoma | Papillary | Mixed | Hürthle cell | Squamous |
|---|---|---|---|---|---|---|---|
| Total | 14 | 5 | 35 | 23 | 28 | 6 | 2 |
| Female | 10 | 4 | 29 | 18 | 23 | 3 | 2 |
| Male | 4 | 1 | 6 | 5 | 5 | 3 | 0 |

TABLE VIII.—*Distribution of Carcinomas According to Age*

| Age in years | 1–10 | 10–19 | 20–29 | 30–39 | 40–49 | 50–59 | 60–69 | 70–79 | 80–89 | Not stated |
|---|---|---|---|---|---|---|---|---|---|---|
| Number of cases | 3 | 3 | 12 | 16 | 22 | 24 | 13 | 11 | 6 | 3 |

tendency to papillary and tubule formation were described as having a mixed structure. Only 4 cases showed definite evidence of multicentric origin.

The series included 6 examples of the non-encapsulated sclerosing tumour in 5 of which there was no evidence of lymphatic metastasis ; in one of these 6 cases there was histological evidence of Graves' disease in the thyroid parenchyma surrounding the tumour. The association between carcinoma and the presence of psammoma bodies which occurred in 36 cases is shown in Table IX and the presence of these structures in a lymphatic metastasis is illustrated (Fig. 7).

TABLE IX.—*Association between Carcinomas and the Presence of Psammoma Bodies*

| Structure | Papillary | Adenocarcinoma | Mixed | Undifferentiated | Giant cell |
|---|---|---|---|---|---|
| Number of cases | 14 | 8 | 11 | 2 | 1 |

In only 6 cases was there definite histological evidence that the carcinoma had originated in a pre-existing colloidal adenoma (Fig. 8). The relationship between carcinoma and associated changes in the surrounding thyroid parenchyma is shown in Table X. Although the series included only 2 examples of squamous celled

TABLE X.—*Relationship Between Carcinomas and Associated Changes in Parenchyma of Surrounding Thyroid*

| Changes in parenchyma of surrounding thyroid | Number of cases | Changes in parenchyma of surrounding thyroid | Number of cases |
|---|---|---|---|
| Squamous metaplasia | 9* | Non-specific changes or material insufficient | 20 |
| Lymphadenoid change | 14 | One adenoma present | 10 |
| Graves' disease | 6* | Several adenomas present | 31 |
| Colloidal hypertrophy | 23 | Complete absence of adenomas | 52 |

* All cases female.

carcinoma focal squamous metaplasia was sometimes observed in tumours having a predominantly different structure (Fig. 9).

In the first group of cases designated " lateral aberrant thyroid " 3 examples showed typical lymphatic metastases clearly arising in each case from an invasive primary carcinoma of the thyroid ; the primary tumour in the fourth example showed the typical features of a non-encapsulated sclerosing tumour. The second group included 3 cases in which minute examination of the thyroid failed to reveal a carcinoma or precancerous lesion. In one of these 3 cases a cystic nodule submitted for examination was erroneously diagnosed by the author as an ectopic papillary cystadenoma of thyroid origin (Fig. 10); further examination of the nodule showed, however, a margin of lymphoid tissue sufficient for its identification as a lymphatic gland (Fig. 11). In another case a series of " lateral aberrant thyroids " were excised over a period of 5 years; these tumours had an identical structure and showed some evidence of very slow growth (Fig. 12).

Although lymphatic glands were submitted for examination in less than one quarter out of the total 113 cases there was evidence of metastasis in 16 cases.

### DISCUSSION

*The statistical relationship between adenoma and carcinoma of the thyroid*

It has been stated by Kearns and Davis (1952) that the solitary thyroid nodule is a clinical myth which cannot be substantiated by gross or microscopic examination; this view receives no support from the present study.

It has been emphasised by Cole, Majarakis and Slaughter (1949) and by Lahey and Hare (1951) that a much higher proportion of carcinomas is found in thyroids containing solitary nodules than in those containing multiple nodules. This concept receives some support from the present study. In this series approximately one half of the total number of carcinomas occurred in thyroids devoid of adenomas but it must be emphasised that over one third of the total number of adenomas occurred as solitary thyroid nodules. When it is considered that the carcinomas were collected over a period of 28 years and that the total number of adenomas filed in the department during this period amount to just over 2000 cases then it is clear that 6·3 per cent of solitary thyroid nodules in the present series were carcinomatous. The clinical diagnosis of carcinoma on any solitary thyroid nodule is, therefore, quite unjustified on its purely isolated nature.

It is furthei apparent that less than one half of the total number of carcinomas in the present series arose in thyroids containing one or more adenomas so that only 3·1 per cent of thyroids containing multiple nodules proved to be carcinomatous. The overall incidence of carcinoma in the present series of cases amounts to 5·4 per cent over a period of nearly three decades; this figure falls within the range previously reported: Crile and Dempsey (1949) 5·6 per cent; Beahrs, Pemberton and Black (1951) 4·8 per cent; Cattell and Colcock (1953) 9·1 per cent; Willis (1961) 4·1 per cent.

It must be emphasised that the multinodular goitre carries a lower risk of malignancy in the general population than is apparent from the figures quoted above. This is due to the fact that in any large published series of surgical cases there is a bias towards the selection of thyroids containing solitary nodules and the rejection of many multinodular goitres arousing no definite clinical suspicion of malignancy. Again Tellem, Stahl and Meranze (1961) have pointed out that a large proportion of clinically diagnosed solitary thyroid nodules have proved on pathological examination to be multinodular lesions, a fact confirmed in the present study; many published estimates of the risk of carcinoma in the solitary thyroid nodule, therefore, will be fallacious.

When the present series of cases is analysed according to age distribution the solitary and multiple adenomas show a maximum incidence in the fifth decade while in the case of the carcinomas the maximum incidence occurs one decade later. In an analysis of the distribution according to sex the benign tumours show a preponderance of female cases in the ratio of 8·0 : 1 while the corresponding ratio for the carcinomas is calculated as only 3·7 : 1.

Only a minority of undifferentiated and giant celled carcinomas in the present series arose in multinodular goitres ; the majority of these tumours were of large size and may have arisen in large pre-existing adenomas as suggested by Piercy (1957) and by Chesky, Hellwig and Welch (1960) although the present study provides no pathological evidence for this view.

It has been stated by Means (1948) and by Piercy (1956) that the majority of thyroid carcinomas arise in pre-existing adenomas while the opposite view has been taken by Meissner and McManus (1952), Sloan (1954) and Lindsay (1960). The former view is usually based on clinical evidence but in this connection it has been pointed out by Pemberton (1939) that a long clinical history in the case of an apparently stationary thyroid nodule is not definite proof of an origin in an adenoma. The latter view has a histological basis since published reports such as the one of Meissner and McManus (1952) as well as the present series of cases show a great preponderance of colloidal adenomas and a marked paucity of papillary adenomas ; the existence of the latter neoplasm has even been challenged by Willis (1960). On the other hand the majority of carcinomas are said to have a papillary structure (Meissner and McManus, 1952 ; Woolner, Beahrs, Black, McConahey and Keating, 1961), but in this connection it must be pointed out that the arithmetical discrepancy is more apparent than real since benign tumours form the great majority in any unselected series of cases.

It has been stated by Cole, Slaughter and Rossiter (1945) and Means (1948) that an accurate estimate of this relationship cannot be made since a carcinoma may overgrow its site of origin and obliterate the evidence of any pre-existing lesion. In the present series only 6 cases of carcinoma showed definite histological evidence of an origin in an adenoma, but the series included six adenomas showing foci of atypical epithelial proliferation and six atypical adenomas. Many of the carcinomas were of small size and it is unreasonable to assume that all traces of a pre-existing adenoma had been destroyed. In the present series the predominance of the papillary carcinoma was not as striking as indicated in some reports although almost one quarter of the carcinomas showed a purely papillary structure. On the basis of this pathological study it must, therefore, be clearly stated that only a small proportion of carcinomas show definite evidence of an origin in an adenoma. It is reasonable to accept the atypical adenoma as a definite precancerous lesion while an adenoma containing atypical foci is probably a precancerous lesion.

*The non-encapsulated sclerosing tumour*

This was described by Hazard, Crile and Dempsey (1949) as a distinct entity, comprising a small papillary tumour more easily discerned in a diffusely enlarged thyroid than in a nodular gland. Its capacity for metastasis has been emphasised by Klinck and Winship (1955) and by Hazard (1960). Only one case in the present series showed evidence of lymphatic metastasis and the very favourable prognosis of this tumour in general has been emphasised by Woolner, Lemmon Beahrs, Black and Keating (1960) ; these authors state that any latent capacity for metastasis cannot be determined on a histological basis. It is clear that some relationship exists between this tumour and one variety of " lateral aberrant thyroid " but the present investigation does not support the suggestion by Hazard (1960) that invasive papillary carcinomas generally arise from this more localised type of tumour.

*The Hürthle celled tumour*

The origin of this tumour is still much debated and eleven theories have been listed by Collins (1956). The above name is now well established in the literature although the interfollicular cell in question was first described by Baber (1877) and later by Hürthle (1894) and bears no relationship to the large oxyphil granular epithelial cell described by Askanazy (1898). On the basis of histochemical studies the latter cells are now regarded as hyperactive rather than as involuted or degenerated (Tremblay and Pearse, 1960).

Some authors have considered that carcinomas whose cells show a granular eosinophilic change behave like any other thyroid carcinoma of comparable structure (Sedgwick, 1952) and that this particular feature carries no added significance in benign or malignant epithelial neoplasms of the thyroid (Horn, 1954). It has been suggested by others (Gardener, 1955 ; Marcus and Watt, 1961) that Hürthle celled tumours are usually of low grade malignancy and cellular pleomorphism is of little significance (Horn, 1954). The present series included more carcinomas than adenomas but the total number of cases is too small to permit definite conclusions. Although the precise nature of this tumour remains obscure and histological assessment of malignancy may be difficult (Chesky, Dreese and Hellwig, 1951) it should be retained in any classification until the problems mentioned above have been clarified.

*Squamous metaplasia and the presence of psammoma bodies*

In the present series there was no significant association between carcinoma and the presence of squamous metaplasia in the surrounding thyroid parenchyma. This finding is in agreement with the views of other authors (Klinck and Menk, 1951 ; Bullock, Hummer and Kahler, 1952).

The distribution of psammoma bodies in the present series is such that their presence makes a diagnosis of carcinoma almost certain although their origin remains obscure. The same conclusion has been reached by several authors (Crile and Fisher, 1953 ; Underwood, Ackerman and Eckert, 1958 ; Batsakis, Nishiyama and Rich, 1960 ; Lindsay, 1960).

*Lymphadenoid change and Graves' disease*

It has been stated by Dailey, Lindsay and Skahen (1955) that there are significant statistical relationships between the incidence of thyroid carcinoma and Hashimoto's disease and between thyroid adenoma and Hashimoto's disease, but that there is no evident relationship between adenoma and carcinoma in thyroid glands affected by the Hashimoto process. The association between carcinoma and chronic thyroiditis and Hashimoto's disease has been noted by several authors (Lindsay, Dailey, Friedlander, Yee and Soley, 1952 ; Pollock and Sprong, 1958 ; Shands, 1960 ; Schlicke, Hill and Schultz, 1960). Two cases were reported by Crile and Fisher (1953) in which a diffuse lymphocytic infiltrate indicative of thyroiditis occurred simultaneously with a diffusely infiltrating paplilary carcinoma; their photographs of carcinomatous infiltration are not, however, entirely convincing.

The present study gives no real support to the views of the above-mentioned authors. There are in the departmental files only 255 examples of lymphadenoid goitre collected over a period of 27 years including both necropsy and surgical material and it is clear that the relationship between the fairly rare lymphadenoid goitre and the very common thyroid adenoma may be purely coincidental. It is of great interest that almost the exact numerical relationship between the adenomas and carcinomas in the present series as a whole is preserved in the presence of lymphadenoid change. It has been claimed by Greene (1957) that there has been a recent increase in the incidence of thyroid carcinoma while at the same time the incidence of Hashimoto's disease is said to have increased (McConahey, Woolner, Black and Keating, 1959). Both these findings may, however, be explained by the recent increase in the amount of surgery undertaken as suggested by Willis (1961) when discussing the incidence of carcinoma.

It has been frequently stated that carcinoma of the thyroid and Graves' disease are two conditions which are only very rarely associated. Thus Pemberton and Black (1948) showed that in a collected series of 1310 cases of thyroid carcinoma only 1·75 per cent showed associated Graves' disease while Sokal (1954) found that thyroid carcinona occurred in only 0·15 per cent of 13,868 patients, affected by Graves' disease. The present series shows a rather more common association between Graves' disease and carcinoma and it is clear that the presence of thyrotoxicosis confers no absolute immunity to the development of carcinoma.

*The multicentric tumour*

In the present series only 4 examples (3·5 per cent) of multicentric origin were found out of a total of 113 cases of thyroid carcinoma. This figure is substantially lower than those reported by other authors : Sloan (1954) 10·6 per cent ; Underwood, Ackerman and Eckert (1958) 32 per cent ; Black, Kirk and Woolner (1960) 20 per cent. The discrepancy may be explained by the fact that a minute histological examination of the thyroid gland was only carried out in the present series of cases when a " lateral aberrant tumour " was discovered and a primary carcinoma of the thyroid was, therefore, suspected. It is clear, however, that minute carcinomatous foci can only be recognised histologically and that more exhaustive examinations of each thyroid gland will yield higher figures.

*The lateral aberrant thyroid*

Some embryologists have suggested that the developing thyroid gland may receive lateral contributions from the fourth or fifth branchial pouches (Weller, 1933 ; Norris, 1937 ; Frantz, Forsythe, Hanford and Rogers, 1942) and this conception has been used to explain the presence of " lateral aberrant thyroids " (Frantz *et al.*, 1942). It must be stated, however, that other workers (Hamilton, Boyd and Mossman, 1945 ; Keith, 1948) have not been able to confirm an embryological basis for the presence of thyroid tissue in a lateral cervical position. The " lateral aberrant thyroid " was accepted by Crile (1939) as a distinct entity but in a later article (Crile, 1947) he was converted to the view that all these tumours are in fact lymphatic metastases from thyroid carcinomas. Webster and Howard

(1954) have suggested that an analogy exists between the " lateral aberrant thyroid " and the parotid adenolymphoma while Melzi and Pagliano (1961) have interpreted 2 thyroid tumours as examples of adenolymphoma ; these tumours have a common clinical feature in their slow rate of growth and they show at least a superficial structural resemblance. The parotid adenolymphoma was thought by several authors to be derived from heterotopic salivary gland rests situated in lymph glands adjacent to or in the parotid gland (Albrecht and Arzt, 1910 ; Harris, 1937 ; Martin and Ehrlich, 1944).

In the present series of cases the first small group were clearly lymphatic metastases from a primary carcinoma of the thyroid but in the second group of 3 cases an exhaustive examination of the thyroid gland failed to reveal a primary tumour. In the face of clinical and histological evidence of very slow growth it is unreasonable to accept these as lymphatic metastases and the analogy mentioned above is worthy of consideration.

### SUMMARY

Approximately one half of the total number of carcinomas occurred in thyroids devoid of adenomas but it must be emphasised that over one third of the total number of adenomas occurred as solitary thyroid nodules. In the present series 6·3 per cent of solitary thyroid nodules were carcinomatous and 3·1 per cent of thyroids containing multiple nodules proved to be carcinomatous.

A large proportion of clinically diagnosed solitary thyroid nodules proved on pathological examination to be multinodular lesions.

The solitary and multiple adenomas showed a maximum incidence in the fifth decade while in the case of the carcinomas the maximum incidence occurred one decade later.

The adenomas showed a preponderance of female cases in the ratio of 8·0 : 1 while the corresponding ratio for the carcinomas was 3·7 : 1.

Only a minority of undifferentiated and giant celled carcinomas in the present series arose in multinodular goitres.

Only a small proportion of carcinomas showed definite evidence of an origin in an adenoma. It is reasonable to accept the atypical adenoma as a definite precancerous lesion, while an adenoma showing atypical foci is probably a precancerous lesion.

The " non-encapsulated sclerosing tumour " is probably a distint entity which carries a favourable prognosis.

There is no evidence that squamous metaplasia or lymphadenoid change in the thyroid parenchyma are precancerous lesions.

The presence of psammoma bodies may for practical purposes be taken as evidence of malignancy ; they are most frequently encountered in papillary carcinoma.

It is clear that the group of cases classified under the heading of " lateral aberrant thyroid " is a heterogenous one. Some of these are examples of lymphatic metastasis from primary carcinomas of the thyroid ; in other cases, however, no primary tumour can be identified and in the consideration of their aetiology it is worth remembering the analogy between this tumour and the parotid adenolymphoma.

My thanks are due to my wife for help with the checking of references and to Dr. S. Robinson for reading the paper. I am indebted to Mr. G. W. Moore for the photomicrographs and to Miss H. Pallan for secretarial work.

### REFERENCES

ALBRECHT, H. AND ARZT, L.—(1910) *Frankfurt. Z. Path.*, **4**, 47.
ASKANAZY, M.—(1898) *Dtsch. Arch. klin. Med.*, **61**, 118.
BABER, E. C.—(1877) *Phil. Trans.*, **166**, 557.
BATSAKIS, J. G., NISHIYAMA, R. H. AND RICH, C. R.—(1960) *Arch. Path.* (*Lab. Med.*), **69**, 493.
BEAHRS, O. H., PEMBERTON, J. DE J. AND BLACK, B. M.—(1951) *J. clin. Endocrin.*, **11**, 1157.
BLACK, B. M., KIRK, T. A., Jr. AND WOOLNER, L. B.—(1960) *Ibid.*, **20**, 130.
BULLOCK, W. K., HUMMER, G. J. AND KAHLER, J. E.—(1952) *Cancer, Philad.*, **5**, 966.
CATTELL, R. B. AND COLCOCK, B P.—(1953) *J. clin. Endocrin.*, **13**, 1408.
CHESKY, V. E., DREESE, W. C. AND HELLWIG, C. A.—(1951) *Ibid.*, **11**, 1535.
*Idem*, HELLWIG, C. A. AND WELCH, J. W.—(1960) *Amer. J. Surg.*, **99**, 857.
COLE, W. H., MAJARAKIS, J. D. AND SLAUGHTER, D. P.—(1949) *J. clin. Endocrin.*, **9**, 1007.
*Idem*, SLAUGHTER, D. P. AND ROSSITER, L. J.—(1945) *J. Amer. med. Ass.*, **127**, 883.
COLLINS, D. C.—(1956) *Arch. Surg., Chicago*, **73**, 228.
COPE, O., DOBYNS, B. M., HAMLIN, E., Jr. AND HOPKIRK, J.—(1949) *J. clin. Endocrin.*, **9**, 1012.
CRILE, G., Jr.—(1939) *Surg. Gynec. Obstet.*, **69**, 39.—(1947) *Ibid.*, **85**, 757.
*Idem* AND DEMPSEY, W. S.—(1949) *J. Amer. med. Ass.*, **139**, 1247.
*Idem* AND FISHER, E. R.—(1953) *Cancer, Philad.*, **6**, 57.
DAILEY, M. E., LINDSAY, S. AND SKAHEN, R.—(1955) *Arch. Surg., Chicago*, **70**, 291.
DONIACH, I.—(1960) ' Recent Advances in Pathology.' London (J. & A. Churchill, Ltd.), 7th ed., p. 255.
FRANTZ, V. K., FORSYTHE, R., HANFORD, J. M. AND ROGERS, W. M.—(1942) *Ann. Surg.*, **115**, 161.
GARDNER, L. W.—(1955) *Arch. Path.* (*Lab. Med.*), **59**, 372.
GRAHAM, A.—(1924) *Surg. Gynec. Obstet.*, **39**, 781.
GREENE, R.—(1957) *Ann. R. Coll. Surg. Engl.*, **21**, 73.
HAMILTON, W. J., BOYD, J. D. AND MOSSMAN, H. W.—(1945) ' Human Embryology', Cambridge (W. Heffer & Sons, Ltd.), p. 163.
HARRIS, P. N.—(1937) *Amer. J. Path.*, **13**, 81.
HAZARD, J. B.—(1960) *Lab. Invest.*, **9**, 86.
*Idem*, CRILE, G., Jr. AND DEMPSEY, W. S.—(1949) *J. clin. Endocrin.*, **9**, 1216.
*Idem* AND KENYON, R.—(1954) *Arch. Path.* (*Lab. Med.*), **58**, 554.
HORN, R. C., Jr.—(1954) *Cancer, Philad.*, **7**, 234.
HÜRTHLE, K.—(1894) *Pflug. Arch. ges. Physiol.*, **56**, 1.
KEARNS, J. E. AND DAVIS, H., Jr.—(1952) *Arch. Surg., Chicago*, **64**, 622.
KEITH, A.—(1948) ' Human Embryology and Morphology '. London (Edward Arnold & Co.), 6th ed., p. 378.
KLINCK, G. H. AND MENK, K. F.—(1951) *Milit. Surg.*, **109**, 406.
*Idem* AND WINSHIP, T.—(1955) *Cancer, Philad.*, **8**, 701.
LAHEY, F. H. AND HARE, H. F.—(1951) *J. Amer. med. Ass.*, **145**, 689.
*Iidem* AND WARREN, S.—(1940) *Ann. Surg.*, **112**, 977.
LINDSAY, S.—(1960) ' Carcinoma of the Thyroid Gland.' Springfield, Illinois. (C. C. Thomas).
*Idem*, DAILEY, M. E., FRIEDLANDER, J., YEE, G. AND SOLEY, M. H.—(1952) *J. clin. Endocrin.*, **12**, 1578.

MARCUS, R. AND WATT, J.—(1961) *Brit. J. Surg.*, **48**, 667.
MARTIN, H. AND EHRLICH, H. E.—(1944) *Surg. Gynec. Obstet.*, **79**, 611.
MCCONAHEY, W. M., WOOLNER, L. B., BLACK, B. M. AND KEATING, F. R., Jr.—(1959) *J. clin. Endocrin.*, **19**, 45.
MEANS, J. H.—(1948) ' The Thyroid and its Diseases '. Philadelphia, London, Montreal. (J. B. Lippincott Co.) 2nd ed., p. 454.
MEISSNER, W. A. AND MCMANUS, R. G.—(1952) *J. clin. Endocrin.*, **12**, 1474.
MELZI, M. AND PAGLIANO, L.—(1961) *Tumori*, **47**, 152.
NORRIS, E. H.—(1937) *Contr. Embryol. Carneg. Instn*, **26**, 247.
PEMBERTON, J. DE J.—(1939) *Surg. Gynec. Obstet.*, **69**, 417.
*Idem* AND BLACK, B. M.—(1948) *Surg. Clin. N. Amer.*, **28**, 935.
PIERCY, J. E.—(1956) *Proc. R. Soc. Med.*, **49**, 174.—(1957) *Post Grad. med. J.*, **33**, 346.
POLLOCK, W. F. AND SPRONG, D. H., Jr.—(1958) *West. J. Surg.*, **66**, 17.
SCHLICKE, C. P., HILL, J. E. AND SCHULTZ, G. F.—(1960) *Surg. Gynec. Obstet*, **111**, 552.
SEDGWICK, C. E.—(1952) *Lahey Clin. Bull.*, **8**, 25.
SHANDS, W. C.—(1960) *Ann. Surg.*, **151**, 675.
SLOAN, L. W.—(1954) *J. clin. Endocrin.*, **14**, 1309.
SOKAL, J. E.—(1954) *J. Amer. med. Ass.*, **154**, 1321.
TELLEM, M., STAHL, T. AND MERANZE, D. R.—(1961) *Cancer, Philad.*, **14**, 67.
TREMBLAY, G. AND PEARSE, A. G. E.—(1960) *J. Path. Bact.*, **80**, 353.
UNDERWOOD, C. R., ACKERMAN, L. V. AND ECKERT, C.—(1958) *Surgery*, **43**, 610.
WARREN, S.—(1931) *Arch. Path.* (*Lab. Med.*), **11**, 255.
WEBSTER, R. AND HOWARD, R.—(1954) *Aust. N.Z. J. Surg.*, **24**, 1.
WELLER, G. L., Jr.—(1933) *Contr Embryol. Carneg. Instn*, **24**, 93.
WILLIS, J.—(1961) *Brit. med. J.*, i, 1646.
WILLIS, R. A.—(1960) ' Pathology of Tumours '. London (Butterworth & Co.), 3rd ed., p. 606.
WOOLNER, L. B., BEAHRS, O. H., BLACK, B. M., MCCONAHEY, W. M. AND KEATING, F. R. Jr.—(1961) *Amer. J. Surg.*, **102**, 354.
*Idem*, LEMMON, M. L., BEAHRS, O. H., BLACK, B. M. AND KEATING, F. R. Jr.—(1960) *J. clin. Endocrin.*, **20**, 89.